# A qualitative examination of the usability of a digital cognitive behavioral therapy for insomnia program after stroke

**DOI:** 10.1080/02699052.2022.2034182

**Published:** 2022-02-02

**Authors:** Tom Smejka, Alasdair L Henry, Catherine Wheatley, Colin A Espie, Heidi Johansen-Berg, Melanie K Fleming

**Affiliations:** a Wellcome Centre for Integrative Neuroimaging, FMRIB, Nuffield Department of Clinical Neurosciences, University of Oxford, Oxford, UK; b Big Health Ltd, London, UK; c Sleep & Circadian Neuroscience Institute, Nuffield Department of Clinical Neurosciences, University of Oxford, Oxford, UK

**Keywords:** CBT, feasibility, interview, usability, recovery

## Abstract

**Objective:**

Sleep is commonly impaired after stroke. Cognitive Behavioral Therapy for Insomnia (CBT-I) is the first-line recommended treatment for sleep difficulty. “Sleepio” is a digital CBT-I program, allowing delivery of this treatment at scale. However, Sleepio has not yet been tested specifically in people with stroke. Before doing so, we wanted to explore the experience of people with stroke using the program, and potential barriers to completion.

**Method:**

Community dwelling survivors of stroke (n = 11, 41–78 years of age, 6 male) were given access to Sleepio. Participants discussed their experiences with the program during a semi-structured interview, which was analyzed using thematic analysis.

**Results:**

We found four common themes: (1) positive and negative experiences impacted engagement with the program, (2) motivation to follow the program was proportional to perceived severity of sleep problem, (3) impractical advice for people with stroke, (4) difficulty operating the program.

**Conclusion:**

Sleepio can be used by some people at the chronic stage of stroke. However, some barriers to completion were highlighted, and not all suggestions were deemed practical for everyone. We therefore suggest possible adaptations which may make the program more easily usable and engaging for survivors of stroke with varying impairments.

## Introduction

Stroke, a type of acquired brain injury that occurs when blood supply to the brain is interrupted, is a leading cause of disability worldwide (1). Survivors of stroke commonly experience disrupted sleep (2,3), with both self-reported and actigraphy measures showing poorer sleep for people with stroke than age-matched controls (4). A recent systematic review estimates the prevalence of insomnia at 15–60% (5). Poor sleep is frequently accompanied by depression and anxiety (5), and may impact on engagement in activities of daily living (6) and quality of life long-term after stroke (7). It is therefore important to investigate treatments to improve sleep in this population.

Cognitive behavioral therapy for insomnia (CBT-I) is the first-line recommended treatment for insomnia (8,9). There are, however, limited studies investigating CBT-I (or adapted CBT-I) after acquired brain injury. Nevertheless, recent research suggests preliminary efficacy at improving sleep quality and insomnia severity in this population (10,11). However, adaptations to standard CBT-I techniques may be required for some due to complications arising from the lesion, such as reduced mobility (11), as well as typically older age which may impact on ability to travel to appointments and comprehend the supplied information (12). Widespread access to in-person CBT-I is typically limited due to a lack of trained professionals and geographical distance (13). Digital CBT-I (web and/or mobile applications) provides an effective option for increasing access to this treatment to meet the demand (14).

Sleepio (www.sleepio.com) is a fully automated digital CBT-I program. Large effects on a range of sleep outcomes across diverse populations have been demonstrated (15–26). However, to our knowledge, digital CBT-I has yet to be examined in people with stroke. As many people experience long term difficulties with movement, language and cognition after a stroke, a digital program may need to be adapted in order to accommodate these difficulties.

We therefore sought to undertake a qualitative study to explore the experience of people with stroke using Sleepio, to gain an indication of usability and guide the design of future randomized controlled trials. Specifically, we aimed to examine 1) the general experience of using the program and 2) whether there were any barriers to completion.

## Methods

### Participants

A convenience sample of participants was recruited through stroke user group meetings and a database of past research participants. Inclusion criteria were: aged ≥18 years, >3 months post-stroke, interest in improving sleep. Exclusion criteria were: current, frequent travel between time-zones, current ongoing shift work. We chose to include people with stroke who did not necessarily present with insomnia, as we felt that they would still have valuable contributions regarding usability of the program in relation to post-stroke difficulties, and a recent study suggests that Sleepio is similarly effective and acceptable in people with sub-clinical insomnia (27).

Twenty people with stroke were emailed the participant information sheet and invited to participate; 2 were ineligible (1 had no interest in improving sleep, 1 was unable to provide consent), 5 didn’t respond to the invitation, 2 declined (1 due to caring responsibilities, 1 didn’t want to take part) and 11 provided written informed consent. A maximum variation sampling approach was used with a focus on finding participants of different ages, stroke outcomes and self-reported familiarity with digital technology. Data collection took place over 7 months, and recruitment stopped once data saturation was reached. The study was approved by the local Research Ethics Committee (R61184/RE001).

### Procedure

Participants completed two visits (baseline and follow-up), at the University or in their homes (based on participant preference). At the baseline visit, they completed the questionnaires then watched a 2-minute introductory video, created an account (with a code that enabled researchers to track progress) and completed the first digital CBT-I session. They then used the Sleepio program at home before returning for the follow up interview.

#### Assessments

Participants completed the Sleep Condition Indicator (SCI-8 (28)), to determine self-reported sleep quality. The SCI is scored from 0 to 32, where lower values are indicative of greater insomnia symptoms and scores ≤ 16 indicate probable insomnia. We also used the Montreal Cognitive Assessment (MoCA (29)) at baseline to gain an indication of cognitive, language and perceptual difficulties which may influence ability to use the program. The MoCA is scored from 0 to 30, and higher values indicate fewer cognitive difficulties.

#### During the program

Sleepio is delivered through a web-based platform or iOS app and is structured around 6 CBT-I sessions (each ~20 minutes). Sessions are available 7 days after completing the previous session. Content is based on cognitive (e.g., cognitive restructuring, paradoxical intention etc.) and behavioral (e.g., stimulus control therapy, sleep restriction therapy and relaxation) techniques, and is delivered by an animated therapist “The Prof.” Treatment is personalized based on questionnaire responses and daily sleep diaries.

A researcher (TS) contacted participants weekly to monitor for adverse effects and to ask how they were finding the program (Table 1). Responses were used to help guide the discussion at the follow up interview, but were not recorded or analyzed specifically. TS also contacted participants when more than four days had passed without accessing an available session to prompt continuation and provide assistance when needed. Although this is a deviation from how Sleepio would ultimately be used, it was felt to be important to ensure that all issues with program use were identified and could be discussed at the final interview.

**Table 1.** Weekly checkup questions

How are you finding the sessions?
How are you finding keeping the diary?
Have you made any changes suggested by the program? Does the weekly schedule suit you? How difficult are you finding it to keep up with the program (scale of 1–10)?

#### Follow-up interview

Following completion of Sleepio (or earlier if they did not want to complete) participants attended a face-to-face semi-structured, audio recorded interview (at the University or in their home, depending on participant preference). The same researcher (TS) conducted all participant interactions in an attempt to maximize participant comfort and openness during interview. The interviewer (TS) is a male postgraduate research assistant with an undergraduate degree in Psychology who was completing this study as part of a Master’s degree in Clinical Neuroscience. He has worked extensively with people following stroke and his research interests are focused around seeking to understand recovery and rehabilitation after stroke. The topic guide included questions aiming to explore experience and ease/difficulty of usage (Table 2). The first participant acted as the pilot for the topic guide, but no changes were deemed necessary prior to continuation of the study. Follow-up probes were used to encourage elaboration on points noted during the weekly contacts or if they mentioned elements that had not been addressed by the structured questions. In an attempt to reduce social desirability and positivity bias the researcher consciously avoided positive framing of questions. Participants were told that TS was not affiliated with Sleepio, and that his goal was to understand their experience of the program to help guide future studies.

**Table 2.** Semi-structured interview topic guide

How did you find using the Sleepio program?
Was there anything you found particularly difficult?
Did you find it easier to use the phone or laptop/ why did you use the modality you used?
Were you able to remember all the different times asked for in the diaries? Did you find it difficult?
How motivated were you to stick with the program?
Do you feel like it helped? Have you noticed a change?
Did you learn anything new about the way we sleep?
What was your favorite thing about the program?
What was your least favorite thing?
Were any problems you found with the program related to your stroke, difficulty using the program/ difficulty remembering to complete the sessions?
Would you suggest anything to change within the program to make it better or easier to use?
Did you use all the functions: diary, checklist etc. if so, were they easy to use/ helpful? If not, why not?
Did you use the library articles or community?
Were you able to implement any of the changes?
Do you have anything else you would like to talk about that we touched on, or do you have any questions for me?

### Analysis

Interview data were analyzed using a reflexive thematic analysis approach as outlined by Braun and Clarke (30), following six phases: familiarization, generating initial codes, searching for themes, reviewing themes, defining and naming themes, and producing a report. An inductive approach was used as there were no preexisting reports on the usage of Sleepio by people with stroke. Audio files were transcribed verbatim by a researcher (TS). Transcripts were not provided to participants, and we did not seek their feedback on the findings. Transcription and initial coding were conducted continuously during the study, while participant recruitment was ongoing, but creation of the themes occurred after all data collection had stopped.

A second researcher (MF) coded four transcripts, chosen pseudorandomly (i.e. one from the first 3 participants, one from the last 3 participants and the remainder from the middle participants). If discrepancies were found, the two researchers discussed this and agreed on a consensus, adapting themes if required.

## Results

Table 3 shows the participant demographics, MoCA and SCI scores. Based on the baseline SCI score, five participants would meet the criterion for probable insomnia disorder. Three participants chose to have a partner help them, who also attended the qualitative interview. One of these participants had a low MoCA score, suggestive of some cognitive impairments. The other two had MoCA scores comparable to other participants who did not seek to have assistance. The researcher (TS) knew seven of the participants from previous studies. The remaining participants were met in person for the first time at the baseline assessment.

**Table 3.** Participant characteristics, time to complete Sleepio, and interview duration

Participant	Age (years)	Sex	Time Since Stroke (mo)	MoCA (max 30)	Baseline SCI (max 32)	SCI at follow up interview (max 32)	Days to Complete Program	Number of CBT-I sessions completed	Interview Duration (mins)
1	54	F	242	27	9	16	54	6	49
2	41	M	107	26	28	31	92	6	40
3	54	F	164	25	9	22	55	6	39
4	78	F	52	24	31	31	53	6	17
5	76	M	18	10	30	25	N/C	3	48
6	70	M	63	29	17	28	114	6	29
7	60	F	128	26	16	18	104	6	27
8	78	M	160	25	23	30	98	6	52
9	72	M	11	26	18	13	N/C	4	43
10	53	M	5	24	12	9	45	6	75
11	59	F	63	28	13	27	43	6	43

MoCA = Montreal Cognitive Assessment (higher values indicate better cognition), SCI = sleep condition indicator (higher values indicate better self-reported sleep quality). Mo = months. N/C = not completed.

**Figure 1.**
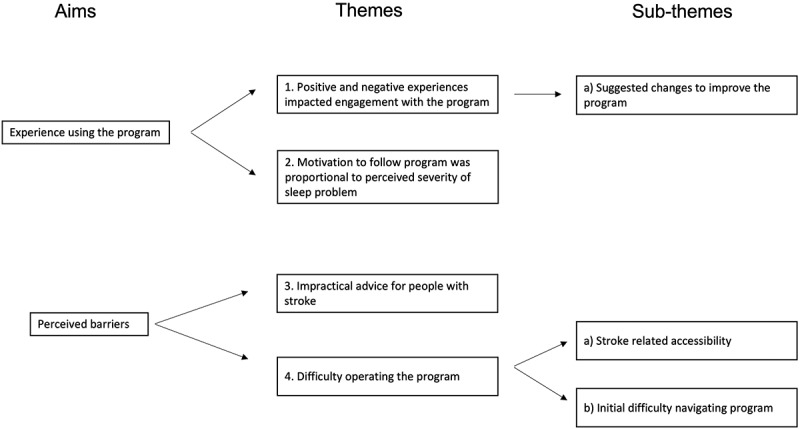
Coding tree.

All participants completed the final interview, including the two who did not finish the Sleepio program. Interviews lasted 42 minutes on average (range 17–75 min; Table 3). Four themes were identified (Figure 1).

### Theme 1: positive and negative experiences impacted engagement with the program

Participants reported feeling positive and actively engaged with Sleepio when the program was found to be effective.

**P3** “All it took was for one good night sleep and then I was just ‘Oh my god I’ve had a good night’s sleep’ and then I was motivated.”

**P11** “I tried every technique under the sun and nothing else had worked … well this did.”

Participants also reported feeling more in control of their sleep:

**P7** “If you’re having trouble it makes you feel a bit more in control of what’s happening to you.”

Even in cases where participants reported not having much of a sleep problem, enjoyment was mentioned in regards to the educational nature. Similarly, the partners aiding participants often commented on their own enjoyment:

**P4** “Very informative actually, good ideas, very good ideas … I enjoyed the whole lot.”

**P5 (Partner)** “We didn’t use the sessions [with the Prof] enough. They were really good, we really liked those.”

Once familiar with the program, participants reported daily usage becoming part of their routine.

**P3** “The sleep diary wasn’t a problem at all … It’s just one of those other apps you check in the morning.”

However, the inclusion of advice that some participants found to be impractical or unfeasible led to feelings of disconnect at times, or feeling that the course might not be for them.

**P1** “I just didn’t feel it was really aware of, of the sort of living situation that people with disabilities have.”

It was frequently mentioned that hearing advice that didn’t apply to them was frustrating:

**P6** “The way they spoke as if most people’s problems was thinking about not sleeping that’s not my case. I can’t sleep because my mind’s active on thinking about completely different things.”

**P8** “It assumes you’re in perfect, in inverted commas ‘health’ all the time but when I was ill that seriously upset my routine.”

This disconnect also led to the perception of the program feeling long or being too demanding:

**P3** “There was just too much information in all the sessions and some of it applied to me and some of it didn’t.”

**P1** “I got slightly flippant by the end because it’s like the same question for 30 days in a row.”

Although the design of the program is to mimic personal CBT-I, at times when advice or information was not well suited to the individual, a disconnection was felt quite strongly.

**P1** “I mean, I sort of lost faith in the whole thing. I continued to do my recording because as I said it was a good process for me just to do the recording but it felt they’d lost me.”

**P11** “I came so close to stopping, so close to stopping because I was entirely convinced they were wrong.”

#### Sub-theme 1a: suggested changes to improve the program

Many participants suggested changes that they thought could be beneficial. Most commonly, they wanted to be able to input more information about their situation.

**P10** “he’s asked rhetorically ‘so how are you doing this week?’ … and I thought well actually I wish I could answer that ….”

**P11** “The one big issue that I had with it, is that there was no … ‘why?,’ you know, I don’t get up at 6 o’clock because I want to, but because I have to … ”

Participants also mentioned wanting additional advice in relation to other factors that were affecting their sleep.

**P1** “possible suggestions for managing pain when you’re trying to go to sleep … it didn’t feel as if it came into the app at all.”

### Theme 2: motivation to follow program was proportional to perceived severity of sleep problem

Participants reported some aspects as challenging, and their motivation to follow advice was proportional to their perception of need for improvement. P1 didn’t perceive their sleep difficulties to be problematic enough to fully engage with all of the suggestions, despite having an SCI score indicative of probable insomnia (9 out of 32).

**P1** “I didn’t find them [suggestions to change the room] hugely useful but I guess it’s not that I feel I need radical changes or that I’m so worried about my sleep that I’ll do whatever it takes.”

Sleep Restriction Therapy (SRT) was identified as a challenge. P11, who reported six years of sleep difficulty, felt a strong desire to try to improve despite the challenges:

**P11** “I had to stay up till midnight and it nearly bloody killed me and I’m just sort of thinking ‘this is ridiculous’ but I thought ‘no I said I would do it’ so I forced myself through.”

However P8 reported that by the time they reached the sleep restriction week they felt that their sleep had improved enough that they didn’t need to engage with more challenging techniques:

**P8** “I had got into a routine and was beginning to crack it.”

### Theme 3: impractical advice for people with stroke

Participants reported that advice could be impractical or unsuitable based on their stroke outcomes. Frequently it was reported that advice to avoid daytime naps was problematic.

**P6** “After a stroke you do get more tired … very often I have to have a little snooze during the day … this tends to say ‘oh don’t do that’ … So, I’m not sure if that’s necessarily good advice.”

**P10 (Partner)** “It’s very difficult to control going to bed and getting up at a certain time and staying awake.”

Participants also reported difficulty with with aspects of Stimulus Control Therapy, particularly the suggestion, when struggling to sleep, to get out of bed and go to another room until sleepy. Several participants had concerns about limited mobility:

**P3** “I’m just worried about being a bit too wobbly in the middle of the night … and with my mobility problems I don’t really want to start moving around the house.”

Difficulties were also reported in regards to Progressive Muscle Relaxation (PMR), which involves systematically tensing and relaxing different body parts. This technique was difficult for participants who experience high muscle tone as a result of one-sided weakness.

**P1** “So I can tense and then yeah I mean it is a problem getting it to relax.”

### Theme 4: difficulty operating the program

A variety of difficulties operating the program were reported. These related to difficulties using computer-based programs due to stroke-related impairment, limited computer literacy or due to elements that were not seen as intuitive.

#### Sub-theme 4a: stroke related accessibility

Outcomes of stroke were mentioned as the cause of difficulty for some. Three participants had a partner to assist as they felt their cognitive difficulties meant they would not have been able to access the program without help.

**P5 (Partner)** “You could not have done it alone darling” **P5** “No, indeed, yes. Remember the first time you had to talk to me many times, sometimes even more than 2, 3 times.”

**P8** “Since my stroke I have lost the ability, attitude or what I use to use the computer and I don’t use it now.”

Even in cases where participants were completing Sleepio alone, it was mentioned that the stroke had had an impact:

**P9** “I mean before the stroke I would have thought I was much more competent in operating a program.”

In contrast, even though five participants had one-sided weakness leading to the functional usage of only one hand (determined through observation and discussion), no issues were reported:

**P2** “I have only one hand, sadly, but everything that I could do, could be done with one hand.”

However, there was a potential issue with accessing some of the written parts of the program. P2 noticed that although sessions were largely audio driven, some questions did not have an accompanying audio.

**P2** “Personally I would like to see all audios so the … what’s it called? ‘Think about the typical night of the last month.’ It was readable but not speaking … So I could read that but some people can’t.”

#### Sub Theme 4b: initial difficulty navigating program

Six participants struggled to find the prompt to start the second digital CBT-I session, requiring contact with the researcher. For some, this early delay was a result of not noticing that a new session was available:

**P1** “It didn’t say “click here … it’s assuming a lot that someone should just assume that you know without a prompt.”

P7, using the iOS app, described the fact that she had not noticed the button used to access the sessions when using the iPad as it was usually “greyed out” when filling in the sleep diary:

**P7** “I didn’t really even notice it because when you first get it you press everything to see what it does (laughs) and that didn’t do anything and then whenever I then looked at it, it was always grey and I couldn’t do anything with it.”

One of the common reasons given by the older participants was a lack of computer experience:

**P4** “I’m not very good with computers. Once I’m set up I’m fine but before that I’m a bit confused you know.”

On the other hand, some participants cited their prior experience with computers as one of the reasons they found the program easy to navigate:

**P11** “I use computers all day, every day so I’m pretty tech savvy and I could see that if somebody wasn’t they might get confused … ”

## Discussion

We sought to explore the experience of people with stroke using the digital CBT-I program “Sleepio” and perceptions of usability. There were no adverse events reported and 9 out of 11 participants were able to complete the program. Many participants found Sleepio educationally enjoyable and useful in improving their sleep. However, there were some clear considerations for future studies, especially if seeking to include people with mobility issues, aphasia and/or cognitive impairment.

Although participants discussed their enjoyment and learning, some specific aspects were reported as not being particularly “stroke friendly.” Many participants struggled to stop napping due to fatigue. This difficulty is not necessarily unique to people with stroke (31), but post-stroke fatigue is highly prevalent and debilitating (32). Adapting this advice to recommend reducing the number or duration of naps, and to nap only at particular times of day, may help the feasibility for participants with fatigue and increase the feeling of inclusion and personalization.

We were pleased to find no clear physical issues associated with operating the program for participants with one-sided arm weakness. However, advice to leave the bed when unable to sleep was considered unsafe for participants with reduced mobility. This has also been noted for in-person CBT-I in this population as well as in people with traumatic brain injury (10,11). This could be adapted by advising users to sit on the edge of a bed, if leaving the room is not feasible, or using relaxation techniques such as those implemented in the study by Herron et al. (11). This may still help to break the bed-insomnia association. Similarly, it was noted that progressive muscle relaxation was deemed to be unsuitable for people with high tone (a common complication associated with motor deficits). It is worth mentioning that all participants are provided information on progressive muscle relaxation, however, following this “the Prof” instructs anyone who experiences muscular or joint problems to use autogentic relaxation instead. Progressive muscle relaxation could therefore be avoided if needed.

Only two participants failed to complete the program. One was unable to identify when new sessions were available, even with support from the researcher and a partner. The second participant was delayed by not having internet access whilst traveling. He reported struggling to follow the program upon return. Whilst this completion rate is promising, future larger studies are needed to examine attrition rate in comparison with other populations, given that in the current study the researcher provided additional contact compared with the standard operating of the Sleepio program.

Three participants reported requiring help from a partner to operate the program. This is an important consideration for future studies, suggesting that a wider group of people may be able to use digital CBT-I if given support from a partner or carer, than if studies are restricted to those who can use it alone. Although it is not possible in this study to determine to what extent these difficulties are due to the stroke *per se*, or due to the older age of the participants (the mean age of the sample was over 60 years), further instructional content such as a navigation tutorial to help novice users, and a more obvious prompt when the digital CBT-I sessions become available, could also prove helpful for this population. Sleepio does offer users the option of receiving e-mail and SMS reminders when new sessions are available, however most participants in this study did not opt to have reminders. This may be have been influenced by the knowledge that a researcher would be checking on them, but may also be a result of not knowing that this option was available. Even with additional navigation aids, it should be considered that individuals with more severe cognitive impairments and technological novices may require external assistance or may not benefit to the same extent. In cases where users need additional support, without access to a friend or relative to provide this help, Sleepio offers the option to connect your profile with your clinician. This feature was used in the present study to allow researchers to monitor participant progression. It is conceivable that outside of research, a hybrid approach could be useful in this population, where Sleepio is used in conjunction with input from a clinician where appropriate. A qualitative study examining in-person CBT-I in older adults with co-morbid insomnia and depression also found that participants expressed a desire for a multimodal delivery approach (12).

### Limitations

One limitation of this study is in our choice to include participants who did not report symptoms of insomnia. This may have led to more negative impressions of participant engagement than would have been observed otherwise, as participants may not have felt that certain techniques were warranted if they had mild symptoms. However, given that recent evidence suggests that Sleepio may also be useful in people with more sub-clinical insomnia symptoms (27), we feel that the inclusion of people with varied severities of sleep difficulty and wide ranging outcomes after stroke helped to ensure a breadth of viewpoints. Similarly, we did not exclude participants experiencing symptoms of other sleep disorders. This was partly because this study was focused on the experience of using the program rather than its efficacy. However, for future efficacy studies it will be important to consider excluding participants with other sleep disorders (such as sleep apnea) given that this will likely influence the level to which a participant can benefit from CBT-I.

Another limitation of this study is that all participants were in the chronic stage of stroke. This was intentional, as this is the time when the majority of rehabilitation input has finished, and ongoing consequences may be realized. It was mentioned by a couple of participants that their ability to complete the program was only possible given their recovery level. Therefore, more research is needed early after stroke to determine whether usability differs at this timepoint or whether there are additional barriers to consider.

The researcher (TS) knew seven of the participants from previous studies, which potentially introduces bias. Additionally, it was clear that some participants felt they should continue with the Sleepio program, having agreed to participate in a study, rather than having a desire to complete Sleepio itself. In an attempt to reduce social desirability and positivity bias the researcher consciously avoided positive framing of questions, and participants were told that TS was not affiliated with Sleepio.

### Summary and implications for future research

Participants found Sleepio to be enjoyable and some reported it as being effective in improving their sleep quality. However, there were some consistently reported issues that could put people with stroke at a higher risk for dropout or incompletion, and so the provision of supplementary information alongside the program may prove useful.

Future research should investigate the efficacy of Sleepio in people with stroke as well as potential predictors of treatment response. If effective, providing Sleepio as part of clinical care could assist in increasing general well-being, reducing the risk of further health problems, and improving quality of life.

## Data Availability

Data is available upon reasonable request to the corresponding author (MF).

## Rights retention

This research was funded in whole, or in part, by the Wellcome Trust [Grant number 110,027/Z/15/Z]. For the purpose of open access, the author has applied a CC BY public copyright licence to any Author Accepted Manuscript version arising from this submission.
